# Comorbidities and Molecular Genetics Status in Familial and Nonfamilial Hypercholesterolemia: A Single-Center Study

**DOI:** 10.3390/ijms27031214

**Published:** 2026-01-25

**Authors:** Olga Timoshchenko, Elena Shakhtshneider, Dinara Ivanoshchuk, Valentina Zorina, Pavel Orlov, Sergey Semaev, Yuliya Ragino

**Affiliations:** 1 Institute of Internal and Preventive Medicine—Branch of Institute of Cytology and Genetics, Siberian Branch of Russian Academy of Sciences (SB RAS), –Branch of ICG SB RAS, 175/1 Borisa Bogatkova Str., Novosibirsk 630089, Russia; 2 Federal Research Center Institute of Cytology and Genetics, SB RAS, 10 Prospekt Ak. Lavrentyeva, Novosibirsk 630090, Russia

**Keywords:** familial hypercholesterolemia, cardiometabolic comorbidities, Dutch Lipid Clinic Network, molecular genetic diagnosis, genetic variants

## Abstract

The aim of the study was to characterize the prevalence of comorbidities and molecular genetic status in patients with familial hypercholesterolemia (FH) and non-familial hypercholesterolemia (non-FH). This cross-sectional observational study included 323 patients. Assessments comprised personal and family histories, physical examination, fasting lipid profiling, and molecular genetic testing. Patients with FH were not characterized by an increased prevalence of type 2 diabetes mellitus. In contrast, the non-FH group demonstrated a pronounced cardiometabolic comorbidity profile with a high prevalence of recurrent chronic pancreatitis. Patients with probable or definite FH had a higher prevalence of coronary heart disease and peripheral atherosclerosis, whereas myocardial infarction (MI) was common across all studied groups. Among patients with definite and probable FH, pathogenetic variants were identified in 78.2% and 71.4%, respectively, predominantly in the *LDLR* gene, with one variant in the *APOB* gene. In the possible FH group, pathogenic variants were identified in 46.7% of cases (*LDLR* gene in 64.3% and *APOB* gene in 28.6%). Patients with FH were characterized by a lower prevalence of concomitant cardiometabolic diseases. The high diagnostic yield of genetic testing in the possible FH category (figured Clinic Network score 3–5) suggests that expanding indications for molecular genetic testing to include this patient group should be considered.

## 1. Introduction

Familial hypercholesterolemia (FH) is among the most common inherited metabolic disorders [1]. Data from global registries collected over the past decade suggest that FH may be more prevalent than previously estimated. Variation in reported prevalence across countries likely reflects both population genetic differences and heterogeneity in diagnostic criteria, as well as differences in the organization and scale of screening programs [2].

Familial hypercholesterolemia (FH) is the most common inherited dyslipoproteinemia, caused by genetic defects that impair low-density lipoprotein (LDL) catabolism. This results in pathological LDL accumulation in blood plasma and accelerated atherosclerosis development.

The genetic architecture of FH is predominantly associated with mutations in genes encoding key proteins involved in LDL cholesterol metabolism. Pathogenic variants in the low-density lipoprotein receptor (*LDLR*) gene are identified in the vast majority of cases (approximately 87%). The *LDLR* gene encodes a receptor involved in receptor-mediated endocytosis that binds low-density lipoprotein and facilitates its cellular uptake [1]. Mutations in the *APOB* gene, which encodes apolipoprotein B (the main apolipoprotein of chylomicrons and low-density lipoproteins and the ligand for the LDL receptor), are detected less frequently (up to 10% of cases).

Additionally, pathogenic variants in the *PCSK9* gene encoding a member of the subtilisin-like proprotein convertase family have been identified in FH cases. This gene is expressed in hepatic, intestinal, and renal tissues and plays a critical role in cholesterol and fatty acid metabolism [1]. *PCSK9* gene variants, which regulate LDL receptor activity, account for less than 5% of cases of autosomal dominant familial hypercholesterolemia.

In rare instances (<1%), defects in the *LDLRAP1* gene, which encodes an adaptor protein involved in LDL receptor internalization, are responsible for the disease. The protein encoded by this gene is a cytosolic protein containing a phosphotyrosine binding (PTB) domain that interacts with the cytoplasmic tail of the LDL receptor [1].

Given the high prevalence of heterozygous familial hypercholesterolemia (HeFH; approximately 1:200–1:250), national clinical and genetic screening programs represent a public health priority. In the Russian Federation, the National Society for the Study of Atherosclerosis maintains the Renaissance Observational Registry [3].

Establishing a network of specialized lipid centers is a priority for improving dyslipidemia management. Expert lipid centers are a key component of comprehensive care for patients with complex dyslipidemias [4,5].

Currently, lipid centers are operational in 27 cities throughout Russia. The Novosibirsk City Lipid Center at the Research Institute of Therapeutic and Preventive Medicine, a branch of the Institute of Cytology and Genetics, Siberian Branch of the Russian Academy of Sciences, represents one of the leading centers in Western Siberia and served as the site for this study [3,4,5].

In real clinical practice, physicians managing Lipid Centers frequently encounter patients who do not satisfy the unified clinical diagnostic criteria for familial hypercholesterolemia (FH). The cohort of patients referred to Lipid Centers with suspected FH may encompass:

- Patients with comorbid conditions in whom concomitant diseases and/or cardiovascular risk factors coexist and influence the lipid profile.
- Younger patients who lack awareness of a family history of cardiovascular disease.
- Patients whose lipid profile values do not meet DLCN diagnostic thresholds, yet whose personal and family medical histories document premature cardiovascular disease.

Characterization of patient cohorts with suspected familial hypercholesterolemia (FH), including those who do not meet strict diagnostic criteria, may contribute to improved differential diagnosis and identify additional index patients with FH who remain undertreated due to their failure to satisfy unified diagnostic criteria.

The aim of the study was to characterize the prevalence of comorbidities and the molecular genetic status in patients with familial hypercholesterolemia (FH) and non-familial hypercholesterolemia (non-FH).

The hypothesis of this study is that in real clinical practice, there are patients who do not meet the strict criteria for familial hypercholesterolemia (FH) but may be index patients with FH who do not receive adequate lipid-lowering therapy to achieve target lipid profile values due to their non-compliance with unified diagnostic criteria.

## 2. Results

### 2.1. Clinical Characteristics of Patients with Dyslipidemia

Sex distribution did not differ between FH and non-FH groups (*p* = 0.162); women comprised the majority across all groups (62.2–79.6%; Table 1). Patients in the unlikely FH and non-FH groups were significantly older than those in the possible, probable, and definite FH groups (median [IQR] age: 58.0 [45.0; 63.0], 52.5 [44.0; 61.0], 43.5 [35.0; 56.0], 46.0 [36.0; 54.0], and 46.0 [35.0; 56.0] years, respectively; pairwise post hoc *p*-values: unlikely FH vs. non-FH, *p* = 0.001; unlikely FH vs. possible FH, *p* = 0.009; unlikely FH vs. probable FH, *p* = 0.007; non-FH vs. definite FH, *p* = 0.003; possible FH vs. definite FH, *p* = 0.034). In post hoc analyses, patients with non-FH had a higher body mass index (27.6 [24.5; 29.6] kg/m^2^) and waist circumference (92.0 [78.0; 106.0] cm) than patients with FH (*p* = 0.011 and *p* = 0.045, respectively). There were no significant between-group differences in smoking status, systolic or diastolic blood pressure, or heart rate (all *p* > 0.05).

### 2.2. Comorbidities in Patients with FH and Dyslipidemia

The prevalence of hypertension was significantly higher in the non-FH group than in the probable and definite FH groups (63.4% vs. 35.3% and 43.3%, respectively; *p* = 0.001 and *p* = 0.003; Table 2). In contrast, for coronary artery disease (CAD), the non-FH group had a higher prevalence than the probable FH group (19.5% vs. 9.8%; *p* = 0.001) but a lower prevalence than the definite FH group (36.7%; *p* = 0.003). Peripheral arterial atherosclerosis was most frequently diagnosed in the definite FH group, with a prevalence significantly higher than that in the possible and probable FH groups (73.3% vs. 55.9% and 44.4%, respectively; *p* _2–4_ = 0.029; *p* _3–4_ = 0.004). Notably, the prevalence in the non-FH group (69.5%) was also significantly higher than in the probable FH group (44.4%; *p* _3–5_ = 0.011).

Analysis of comorbidities associated with secondary dyslipidemia showed that T2DM was significantly more prevalent in the non-FH group than in any FH subgroup (24.4% vs. 3.6%, 7.8%, 1.9%, and 6.7%, respectively; overall between-group *p* < 0.001). Chronic pancreatitis with frequent relapses was observed only in the non-FH group (9.8%), with no cases in any FH subgroup (*p* < 0.001). By contrast, the prevalence of hepatobiliary disorders did not differ significantly across groups (*p* values 0.053–0.615).

Alcohol abuse was more common in the non-FH group than in the FH subgroups, reaching statistical significance only in the comparison with the group of the possible FH (*p* _2–5_ = 0.015). A history of malignant neoplasms was recorded in 5.5% and 7.3% of patients in the unlikely FH and non-FH groups, respectively, with no cases in the remaining groups (*p* _1–2_ = 0.021; *p* _2–5_ = 0.008).

**Figure 1:**
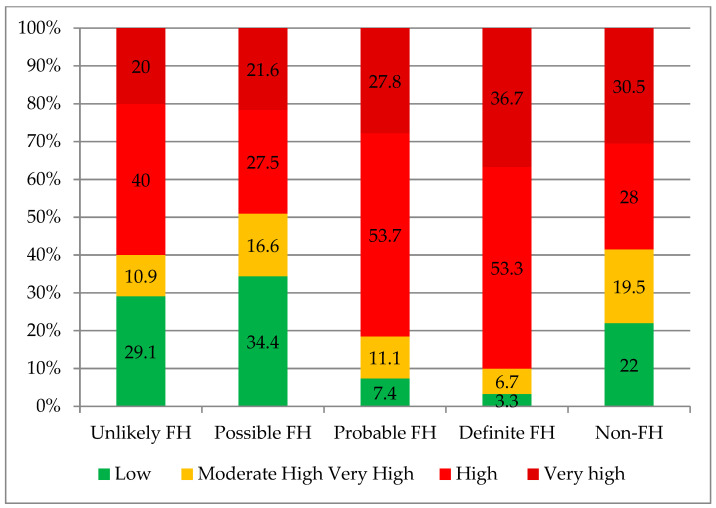
Distribution of cardiovascular risk categories across FH diagnostic groups (overall between-group *p* < 0.001).

Cardiovascular risk stratification showed a predominance of low-risk patients in the unlikely FH, possible FH, and non-FH groups. In contrast, the proportion of patients at high cardiovascular risk was significantly higher in the probable and definite FH groups (overall between-group *p* < 0.001; see Figure 1).

### 2.3. Family History and Lipid Metabolism Parameters in Patients with Dyslipidemia

A positive family history of premature atherosclerotic cardiovascular disease (CVD) was significantly more frequent in patients with definite FH than in all other groups (93.3% vs. 20.0%, 49.0%, 68.5%, and 8.5% in the unlikely, possible, probable, and non-FH groups, respectively; *p* _1–4_, *p* _2–4_, *p* _4–5_ < 0.001; *p* _3–4_ = 0.011). Conversely, the absence of such a history was most common in the unlikely FH group and was significantly more prevalent than in the possible, probable, and definite FH groups (78.2% vs. 51.0%, 31.5%, and 6.7%, respectively; *p* _1–2_ = 0.001; *p* _1–3_, *p* _1–4_ < 0.001; Table 3). Notably, more than one-third of patients in the non-FH group (35.4%) reported being unaware of any CVD in their first-degree relatives. A family history of elevated LDL-C was reported more frequently by patients with definite and probable FH than by those with unlikely or possible FH (57.4% and 56.7% vs. 32.7% and 39.2%, respectively; *p* _1–3_ = 0.019; *p* _1–4_, *p* _2–3_ = 0.046). This prevalence was significantly lower in the non-FH group than in all FH groups (13.4%; *p* _1–5_ = 0.017; *p* _2–5_, *p*_3–5_, *p* _4–5_ < 0.001).

A personal history of early-onset peripheral artery disease was significantly less prevalent in the unlikely FH and non-FH groups than in the possible, probable, and definite FH groups (12.5%, 28.2%, 53.8%, 57.1%, and 63.6% for unlikely FH, non-FH, possible FH, probable FH, and definite FH, respectively; *p* < 0.001). In contrast, the prevalence of early-onset coronary heart disease did not differ significantly across the study groups (*p* = 0.484).

Clinical signs of dyslipidemia showed a strong association with diagnostic certainty. Tendon xanthomas were present in 73.3% of patients with definite familial hypercholesterolemia (FH) and 10.9% with probable FH, whereas xanthelasma occurred in 16.7% and 7.3%, respectively; both were significantly more common than in other groups (*p* < 0.001 for all comparisons). Corneal arcus was observed only among patients with definite (16.7%) or probable (1.8%) FH, with no cases in the remaining groups (*p* < 0.001).

Lipid profiles showed substantially higher total cholesterol (TC) and low-density lipoprotein cholesterol (LDL-C) in patients with probable or definite familial hypercholesterolemia (FH) compared with those with unlikely or possible FH. Median TC (IQR) was 8.5 mmol/L [7.2–9.6] and 9.2 mmol/L [7.8–10.5] in the probable and definite FH groups, respectively, versus 6.6 mmol/L [5.2–7.3] and 7.4 mmol/L [6.4–8.4] in the unlikely and possible FH groups (*p* < 0.001).

**Figure 2:**
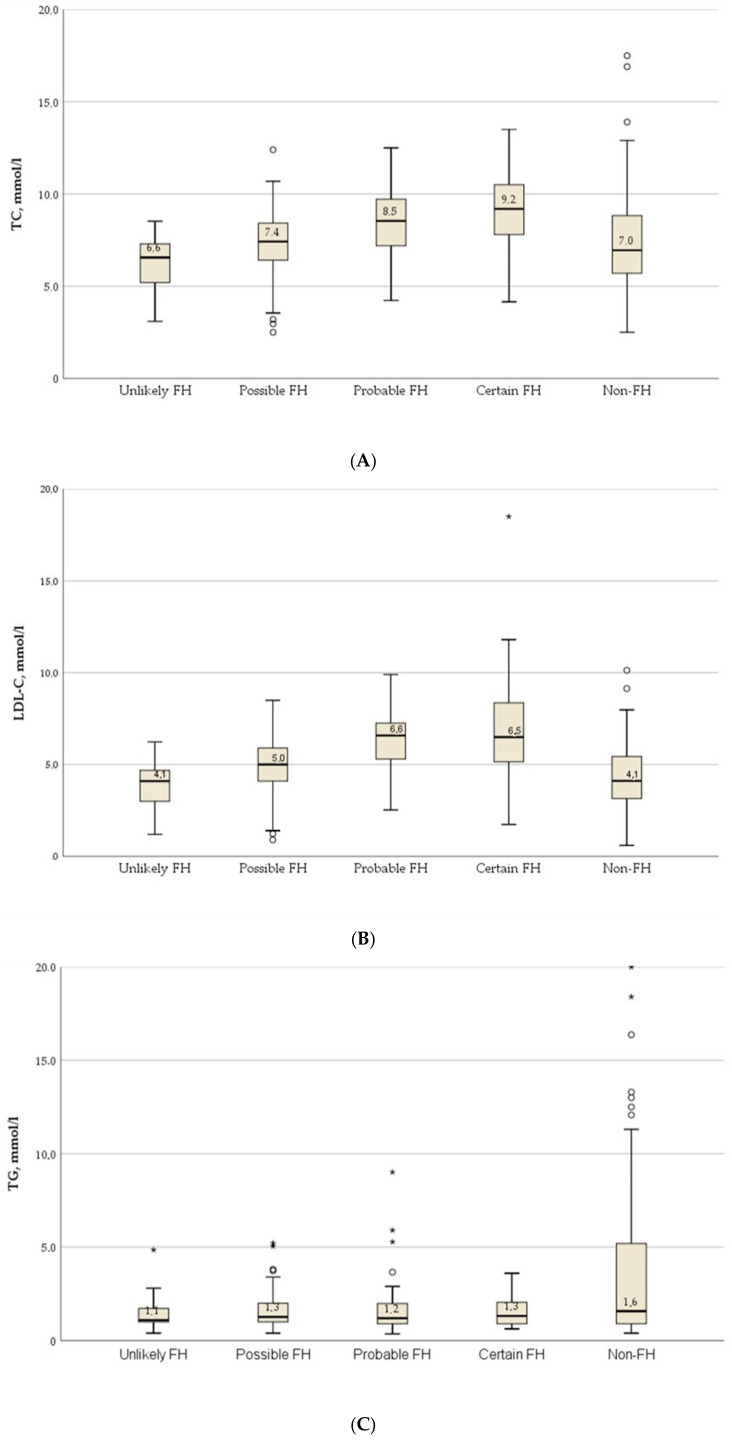
Lipid levels in dyslipidemia groups: (**A**), total cholesterol (TC); (**B**), low-density lipoprotein cholesterol (LDL-C); (**C**), triglycerides (TG). Overall, between-group *p* < 0.001. Box-and-whisker plots are shown: the horizontal line within each box denotes the median; box limits indicate the interquartile range (IQR; 25th–75th percentiles); whiskers extend to the most extreme values within 1.5 × IQR of each quartile; (**A**–**C**) points (dots and asterisks) represent values outside this range (outliers). Dots indicate outliers (>1.5 × IQR) and asterisks indicate extreme outliers (>3 × IQR).

Corresponding median (IQR) LDL-C values were 6.6 (5.3–7.0) and 6.5 (5.2–8.4) mmol/L in the probable and definite FH groups, respectively, versus 4.1 (3.0–4.7) and 5.0 (4.1–5.9) mmol/L in the unlikely and possible FH groups (*p* < 0.001; Figure 2). Triglyceride (TG) concentrations were also significantly higher in the non-FH group than in all FH groups (*p* < 0.001).

### 2.4. Molecular Genetic Diagnostics

According to national clinical guidelines [6,7], the indication for molecular genetic testing is the identification of 6 or more points according to DLCN criteria in a patient.

Among patients with definite FH, pathogenic variants were identified in 78.2% of those molecularly genetically tested, all in the *LDLR* gene. Among patients with probable FH, pathogenic variants were identified in 71.4%, most in the *LDLR* gene and in one in the *APOB* gene. In the group with the possible FH pathogenic variants were identified in 46.7%, distributed as follows: the *LDLR* gene 64.3% and the *APOB* gene, 28.6%. Information about identified pathogenic variants has been added to Supplementary Table S1.

Patients with DLSN scores from 3 to 5, usually, the group with the possible FH, do not receive referrals for molecular genetic testing. Nevertheless, our previous work has shown that testing individuals with DLCN scores from 3 to 5 can identify pathogenic variants in lipid metabolism genes, particularly among younger patients [8,9].

Of the non-FH group pathogenic variant in the *LPL* gene was identified in one patient. The *LPL*-variant carrier had recurrent chronic pancreatitis, type 2 diabetes mellitus, and alcohol use disorder. The lipid profile revealed severe hypertriglyceridemia (66.9 mmol/L), hypercholesterolemia (13.9 mmol/L), and low high-density lipoprotein cholesterol (0.44 mmol/L).

## 3. Discussion

Our study showed that patients with possible, probable, or definite familial hypercholesterolemia (FH) presented for lipidology consultation at a younger age, with group-specific median ages between 43.5 (IQR 35.0–56.0) and 46.0 (IQR 35.0–56.0) years. This aligns with findings from the Kazan Lipid Center reported by Kim Z.F. et al. (2023), where the mean age of FH patients was 48.8 ± 0.7 years (*n* = 127) [10]. By contrast, the nationwide RENESSANS registry reported a higher mean age of 54 ± 13 years (*n* = 1208) [3]. These differences may reflect variation in patient selection and referral patterns or delayed diagnosis in routine clinical practice.

Further supporting our findings, Blokhina et al. (2024) [11] analyzed three independent Russian cohorts—ESSE-Ivanovo (*n* = 1858), ESSE-FH-RF (*n* = 88), and a clinical sample from the biobank of the National Medical Research Center for Therapy and Preventive Medicine (*n* = 1428). Based on molecular genetic testing and lipid profiling, participants were classified into familial dysbetalipoproteinemia (*n* = 29), suspected heterozygous familial hypercholesterolemia (FH; *n* = 61), polygenic hypercholesterolemia (*n* = 49), severe hypercholesterolemia (*n* = 41), and a control group (*n* = 144). Patients with FH (median age 50 [40–61] years) and familial dysbetalipoproteinemia (50 [46–59] years) were significantly younger than those with severe hypercholesterolemia (58 [53–61] years) or polygenic hypercholesterolemia (56 [52–60] years; *p* = 0.027) [11]. The earlier presentation of patients with FH reflects the aggressive nature of the disease and likely heightened clinical suspicion in families with a documented history of FH.

Our analysis revealed statistically significant differences in the pattern of somatic comorbidities between patients with and without FH. The non-FH group showed a pronounced cardiometabolic risk profile, with higher prevalences of arterial hypertension, coronary artery disease, and especially type 2 diabetes mellitus, as well as higher body mass index and waist circumference. They also had a more complex comorbidity burden, including hepatobiliary disorders and behavioral risk factors such as alcohol misuse. By contrast, patients with probable or definite FH had elevated cardiovascular risk driven primarily by dyslipidemia, reflected in a higher prevalence of coronary artery disease and peripheral atherosclerotic disease (particularly in the definite FH subgroup), together with a lower prevalence of concomitant metabolic disorders.

In a systematic review by Vaseghi G et al. demonstrated that among studied comorbidities, FH patients had a greater prevalence of frequently recurring chronic pancreatitis [12]. In our study, the non-FH group exhibited a pronounced cardiometabolic comorbidity profile with a high prevalence of frequently recurrent chronic pancreatitis.

Regarding T2DM, the literature presents inconsistent findings. Some studies report a higher prevalence of diabetes in FH patients, while others indicate the opposite. The systematic review by Vaseghi et al. found that hypertension has been associated with FH, though the prevalence of hypertension varies across FH diagnostic categories. In comparison to the general population, cancer demonstrated lower or similar prevalence in FH patients [12,13].

As expected, these findings suggest that dyslipidemia—particularly hypertriglyceridemia—in non-FH patients likely represents a secondary manifestation of underlying metabolic disturbances [6,14]. Comorbidities may prevent the achievement of the target level of LDL-C when using lipid-lowering therapy [15].

From a clinical perspective, these findings support distinct therapeutic strategies. Patients with FH require early, intensive lipid-lowering therapy, whereas management of non-FH patients should prioritize comprehensive metabolic optimization through weight reduction, increased physical activity, and targeted pharmacological treatment of primary metabolic abnormalities [6,7]. Because patient stratification in our study was based on the Dutch Lipid Clinic Network criteria, it was expected that the definite FH group would show significantly higher proportions and levels of parameters that directly contribute to this score. Specifically, this group had a higher prevalence of a positive family history of CVD and dyslipidemia, more frequent cutaneous stigmata of hypercholesterolemia, and elevated TC and LDL-C concentrations. These findings are consistent with large international studies that have validated these diagnostic criteria [6,7,16,17,18,19].

The high rate of genetically confirmed FH in our cohort—78.2% in the definite group and 46.7% in the possible group among individuals referred for testing—supports the utility of the clinical criteria. Moreover, the substantial proportion of positive genetic results in the possible group (DLCN score from 3 to 5) suggests that extending genetic testing to patients with DLCN scores below 6 may be warranted.

This conclusion is supported by real-world evaluations of FH clinical criteria. In a retrospective cohort of 836 patients with genetically verified FH, Noto et al. (2022) showed that the Dutch Lipid Clinic Network (DLCN) score had modest discriminatory ability for detecting pathogenic variants (AUC 0.662) and performed worse than LDL-C alone (AUC 0.737) [20]. Casula et al. (2018) [21] identified another key limitation: missing data for essential DLCN variables. In their retrospective analysis of 1377 genetically confirmed FH patients (mean age 42.9 ± 14.2 years), 43.4% lacked at least one of eight core parameters in their medical records, and about 10% lacked four or more, most often in the family history and physical examination sections. These omissions systematically lowered calculated scores and, consequently, underestimated FH probability, shifting patients from probable/definite to unlikely/possible categories [21].

The study by Moritz Ferch et al. (2025) [22] also underscores substantial missing data and marked discrepancies between retrospective and initial assessments, leading to poor reproducibility and increased subjectivity of the scoring system. Moreover, an LDL-C threshold of ≥190 mg/dL showed higher sensitivity for detecting genetically confirmed FH than a DLCN score ≥ 6 points [22].

Tada H. et al. demonstrated that molecular genetic analysis confirmed FH in 71.0% of patients with a confirmed diagnosis, 25.9% with a probable diagnosis, 11.7% with a possible diagnosis, and 1.5% with an unlikely diagnosis [23]. These findings confirm that clinical stratification, even when applied to patients with lower scores, serves as a significant predictor of monogenic FH, which, according to current evidence, is critical for appropriate cardiovascular risk stratification.

Notably, genetic confirmation in patients with possible FH not only establishes the etiology but may also be essential for determining cardiovascular risk stratification, LDL-C target values, and treatment intensity.

Limitations. This study was conducted at a specialized lipid center, which likely enriched the cohort for more severe dyslipidemia and a greater comorbidity burden. The cross-sectional design precludes inference about temporality and causality between the observed characteristics and the development of CVD. Residual confounding cannot be excluded, including unmeasured factors such as the duration of and adherence to lipid-lowering therapy, dietary patterns, and physical activity. Patient stratification was based on the DLCN clinical criteria, the sensitivity of which may have been reduced by incomplete medical records, particularly for family history and physical examination findings.

Furthermore, differences in subgroup sizes represent a methodological limitation that may have affected the statistical power of the analysis and should be considered when interpreting these results.

Family history was obtained through patient interviews and may underestimate hereditary predisposition because respondents may be unaware of relatives’ health status, causes of death, or laboratory results. Finally, incomplete coverage of molecular genetic testing—especially among those classified as unlikely FH–precludes a comprehensive assessment of the spectrum and frequency of pathogenic variants in this cohort.

## 4. Materials and Methods

### 4.1. The Study Groups

This cross-sectional observational study enrolled 323 adults aged ≥18 years (*n* = 97 men [30%]; median age 49 years [IQR 38–59]). All participants attended the Novosibirsk City Lipid Center at the Research Institute of Therapeutic and Preventive Medicine, a branch of the Institute of Cytology and Genetics, Siberian Branch of the Russian Academy of Sciences, for specialist cardiological consultation regarding dyslipidemia between January 2020 and December 2024.

The study protocol was approved by the Local Ethics Committee of the Institute of Internal and Preventive Medicine–branch of ICG SB RAS (protocol no. 68, dated 4 June 2019). Informed consent was obtained from each patient.

Indications for referral to the Lipid Center:

1. TC > 10 mmol/L and/or LDL-C > 8.5 mmol/L and/or TG > 11 mmol/L.

2. TC > 7.5 mmol/L and/or LDL-C > 5.0 mmol/L and/or TG > 5.0 mmol/L and/or Lp (a) > 500 mg/L, in combination with a family history of premature atherosclerotic cardiovascular disease (onset before age 50 in men and before age 55 in women).

3. Inadequate response to lipid-lowering therapy, defined as an LDL-C reduction of less than 30% after at least 3 months on a maximally tolerated combination regimen, including cases due to drug intolerance.

4. Personal history of premature atherosclerotic cardiovascular disease (onset before age 40).

5. First-degree relative (parent, sibling, or child) of a patient with a diagnosed inherited atherogenic disorder of lipid metabolism.

### 4.2. Measures and Clinical Data

At the initial consultation, a cardiologist-lipidologist obtained a detailed personal and family history, including early-onset cardiovascular disease and atherosclerotic disease of the cerebral or peripheral arteries (in men under 55 years and women under 60 years), the presence of tendon xanthomas in first-degree relatives, and relatives’ lipid profiles. The physical examination included assessment for stigmata of dyslipidemia, such as xanthelasma palpebrarum, tendon xanthomas, and corneal arcus.

Blood samples for biochemical tests were taken once from the cubital vein in the morning on an empty stomach (12 h after a meal). The fasting lipid profile, measuring total cholesterol (TC), low-density lipoprotein cholesterol (LDL-C), high-density lipoprotein cholesterol (HDL-C), and triglycerides (TG) and blood glucose concentration were determined by enzymatic methods on an automatic biochemical analyzer KoneLab300i (Thermo Fisher Scientific Oy, Vantaa, Finland) with Termo Fisher reagents (Thermo Fisher Scientific Oy, Vantaa, Finland).

To evaluate potential secondary causes of dyslipidemia, thyroid function tests and glycated hemoglobin (HbA1c) were obtained. Its parameters were obtained from patients’ medical records, with analyses performed at various laboratories as part of routine clinical practice.

A blood sample was collected for molecular genetic analysis. According to national clinical guidelines, molecular genetic testing was indicated for patients with a DLCN score greater than 6 points [6]. Subclinical atherosclerosis was assessed by carotid duplex ultrasonography. Cardiovascular risk was estimated using the SCORE2 algorithm or a validated global 10-year cardiovascular risk score.

Management of dyslipidemia followed current clinical guidelines [6].

The likelihood of familial hypercholesterolemia (FH) was assessed using the Dutch Lipid Clinic Network (DLCN) criteria. Based on DLCN scoring, patients were classified into five groups:

Unlikely FH (group 1)–DLCN score < 3; *n* = 55, 17.0%,

Possible FH (group 2)–DLCN score 3–5; *n* = 102, 31.6%,

Probable FH (group 3)–DLCN score 6–8; *n* = 54, 16.7%,

Definite FH (group 4)–DLCN score > 8; *n* = 30, 9.3%,

Non-FH (group 5)–who did not meet the criteria for FH, for example, had high triglyceride levels or low LDL-C levels; *n* = 82, 25.4%.

### 4.3. Genetic Study

DNA was isolated from blood samples using phenol-chloroform extraction [24]. The quantity and quality of extracted DNA were assessed by spectrophotometry using an Epoch microplate spectrophotometer (BioTek Instruments Inc., Winooski, VT, USA). The DNA concentration was ≥50 ng/µL.

For patients meeting criteria for molecular genetic testing, initial analysis consisted of bidirectional Sanger sequencing of the *LDLR* promoter and all coding exons, including flanking splice junctions.

In patients in whom no pathogenic variants were identified in *LDLR*, we performed targeted high-throughput sequencing using a custom panel of 43 genes associated with hypercholesterolemia and disorders of lipid metabolism (including *LDLR*, *APOB*, *PCSK9*, *LDLRAP1*, *CETP*, *LPL*, *HMGCR*, *ABCG5*, *ABCG8*, *APOE*, and *LPA*). The panel encompassed all coding exons and flanking splice junctions. Libraries were prepared with the NimbleGen SeqCap Target Enrichment system (Roche, Basel, Switzerland) and sequenced on the MiSeq platform (Illumina, San Diego, CA, USA), achieving 97% target coverage. Reads were aligned to the human reference genome (GRCh38). Primary processing and variant annotation were performed using the NGS Wizard platform (genomenal.com). All rare variants classified as likely pathogenic or pathogenic were confirmed by automated bidirectional Sanger sequencing.

Variant annotation incorporated assessments of clinical significance based on data from ClinVar and VarSome, as well as evidence from the published literature. Population allele frequencies were annotated using gnomAD v3.1.2 [25] and the RUSeq database. Variants classified in ClinVar or VarSome as benign or likely benign—or those predicted by in silico tools to be benign or likely benign—were excluded from further analysis. The pathogenicity of novel variants was evaluated in accordance with the guidelines of the American College of Medical Genetics and Genomics and the Association for Molecular Pathology (ACMG/AMP) [26].

For patients in whom no pathogenic single-nucleotide or small indel variants were identified, multiplex ligation-dependent probe amplification (MLPA) was performed to detect large deletions or duplications affecting the promoter and exonic regions of the *LDLR* gene. MLPA was conducted with the commercial SALSA MLPA Probemix P062 (MRC-Holland, Amsterdam, The Netherlands), and data were analyzed using Coffalyser.Net software (MRC-Holland; https://www.mrcholland.com; accessed 17 June 2021).

### 4.4. Statistical Analyses

Statistical analyses were performed using IBM SPSS Statistics, version 27.0. Distribution normality was assessed using the Kolmogorov–Smirnov test. Quantitative variables are presented as median (IQR 25th–75th percentiles) due to non-normal distribution.

Between-group differences for these variables were assessed using the Kruskal–Wallis test; when significant, pairwise post hoc comparisons were performed with Dunn’s test and Bonferroni adjustment. Categorical variables are presented as counts (*n*) and percentages (%). Group comparisons for categorical data were conducted using Fisher’s exact test or Pearson’s chi-square test, as appropriate; post hoc analyses used Pearson’s chi-square test with *p*-values adjusted by the Benjamini–Hochberg procedure. A two-sided *p* < 0.05 was considered statistically significant.

## 5. Conclusions

In real-world clinical practice, physicians frequently encounter patients who do not fully satisfy the unified diagnostic criteria for familial hypercholesterolemia (FH), thereby complicating early disease identification and management.

To minimize diagnostic misclassification, clinicians should prioritize complete documentation of all core Dutch Lipid Clinic Network (DLCN) variables, with particular emphasis on comprehensive family history and physical examination findings. Meticulous data collection prevents artificial reduction in the DLCN score and avoids underestimation of FH probability, which would otherwise misclassify patients from definite or probable FH categories into possible or unlikely categories.

Accurate diagnosis of FH in routine clinical practice requires integration of two complementary approaches: application of DLCN clinical criteria combined with careful phenotypic assessment of concomitant metabolic comorbidities. Patients with FH demonstrate a characteristic dyslipidemia-driven atherosclerotic phenotype with elevated cardiovascular risk predominantly attributable to severe lifelong dyslipidemia, manifested by high prevalence of early-onset coronary artery disease and peripheral arterial atherosclerosis in the relative absence of metabolic disturbances such as type 2 diabetes mellitus, abdominal obesity, hepatobiliary dysfunction, or hypertension. In contrast, patients with non-FH dyslipidemia typically exhibit a cardiometabolic syndrome phenotype with multiple metabolic comorbidities. This phenotypic distinction has important therapeutic implications: FH patients require early, intensive, evidence-based lipid-lowering therapy to achieve LDL-C targets; conversely, management of non-FH dyslipidemia should prioritize comprehensive metabolic optimization through weight reduction, increased physical activity, glycemic control, and targeted treatment of primary metabolic disorders.

The finding that pathogenic genetic variants were identified in nearly half of patients with possible FH (DLCN score 3–5) suggests the need to lower the threshold for molecular genetic testing beyond the traditional DLCN ≥ 6 criterion. Genetic testing should be considered for individuals with DLCN scores of at least 3, particularly younger patients presenting with significant dyslipidemia and absence of metabolic comorbidities. Earlier molecular confirmation of FH diagnosis substantially improves the likelihood of achieving guideline-recommended lipid targets and reducing long-term atherosclerotic cardiovascular disease burden. These findings underscore the value of practice-oriented research in refining and optimizing diagnostic algorithms for improved disease differentiation in clinical care.

## Figures and Tables

**Table 1:** Clinical characteristics of patients with dyslipidemia (*n* = 323).

Indicator	FH, *n* = 241				Non-FH (Group 5, *n* = 82)	*p*
	Unlikely (Group 1, *n* = 55)	Possible (Group 2, *n* = 102)	Probable (Group 3, *n* = 54)	Definite (Group 4, *n* = 30)		
Men, *n* (%)	20 (36.4)	27 (26.5)	11 (20.4)	8 (26.7)	31 (37.8)	0.162
Women, *n* (%)	35 (63.6)	75 (73.5)	43 (79.6)	22 (73.3)	51 (62.2)	
Age, years, Me [25; 75]	58.0 [45.0; 63.0]	43.5 [35.0; 56.0]	46.0 [36.0; 54.0]	46.0 [35.0; 56.0]	52.5 [44.0; 61.0]	**<0.001** *p* _1–2_ = 0.001 *p*_1–3_ = 0.009 *p*_1–4_ = 0.007 *p*_2–5_ = 0.003 *p*_3–5_ = 0.034
Smoking status, *n* (%)						0.078
• smokes	2 (3.6)	3 (2.9)	2 (3.7)	4 (13.3)	9 (11.0)	
• never smoked	53 (96.4)	99 (97.1)	51 (94.4)	26 (86.7)	72 (87.8)	
• quit	0 (0)	0 (0)	1 (1.9)	0 (0)	1 (1.2)	
Body mass index, kg/m^2^, Me [25; 75]	23.4 [26.7; 29.4]	24.1 [21.4; 27.3]	23.4 [26.1; 28.8]	23.7 [25.3; 30.3]	27.6 [24.5; 29.6]	**0.011** *p* _1–5_ = 0.001 *p*_2–5_ = 0.001 *p*_3–5_ = 0.001 *p*_4–5_ = 0.001
Waist circumference, cm, Me [25; 75]	76.5 [67.5; 86.5]	78.0 [71.5; 85.0]	77.0 [67.0; 87.0]	82.5 [77.0; 89.5]	92.0 [78.0; 106.0]	**0.045** *p* _1–5_ = 0.007 *p*_2–5_ = 0.008 *p*_3–5_ = 0.007 *p*_4–5_ = 0.038
Systolic blood pressure, mmHg, Me [25; 75]	122.0 [114.0; 130.0]	120.0 [110.0; 130.0]	120.0 [110.0; 130.0]	121.0 [110.0; 130.0]	125.0 [113.0; 137.5]	0.284
Diastolic blood pressure, mmHg, Me [25; 75]	80.0 [74.5; 86.0]	80.0 [72.0; 86.0]	80.0 [70.0; 80.0]	80.0 [70.0; 80.0]	80.0 [75.0; 90.0]	0.111
Pulse, bpm, Me [25; 75]	70.0 [66.0; 75.0]	70.0 [65.0; 79.0]	70.0 [65.5; 79.0]	71.5 [66.0; 77.5]	70.0 [66.0; 79.0]	0.951

**Table 2:** Somatic comorbid conditions in patients with dyslipidemia, *n* (%).

Indicator	FH, *n* = 241				Non-FH (Group 5, *n* = 82)	*p*
	Unlikely (Group 1, *n* = 55)	Possible (Group 2, *n* = 102)	Probable (Group 3, *n* = 54)	Definite (Group 4, *n* = 30)		
**Cardiovascular diseases**						
Arterial hypertension	28 (50.9)	36 (35.3)	18 (33.3)	13 (43.3)	52 (63.4)	**0.001** *p* _2–5_ = 0.001 *p*_4–5_ = 0.003
Coronary heart disease	7 (12.7)	10 (9.8)	11 (20.4)	11 (36.7)	16 (19.5)	**0.012** *p* _2–5_ = 0.001 *p*_4–5_ = 0.003
Of these, revascularization	4 (57.1)	1 (10.0)	5 (45.5)	1 (9.1)	7 (43.8)	0.054
Myocardial infarction	4 (7.3)	7 (6.8)	6 (11.1)	4 (13.3)	8 (9.8)	0.451
Atrial fibrillation	2 (3.6)	0 (0)	0 (0)	0 (0)	1 (1.2)	0.170
Aortic stenosis	0 (0)	1 (1.0)	1 (1.9)	2 (6.7)	0 (0)	0.065
Ischemic stroke	4 (7.3)	3 (2.9)	5 (9.3)	0 (0)	4 (4.9)	0.292
Peripheral arterial disease	34 (61.8)	57 (55.9)	24 (44.4)	22 (73.3)	57 (69.5)	**0.022** *p* _2–4_ = 0.029 *p*_3–4_ = 0.004 *p*_3–5_ = 0.011
**Endocrine diseases and conditions**						
Type 2 diabetes mellitus	2 (3.6)	8 (7.8)	1 (1.9)	2 (6.7)	20 (24.4)	**<0.001** *p* _1–5_ = 0.006 *p*_2–5_ = 0.006 *p*_3–5_ < 0.001 *p*_4–5_ = 0.037
Impaired glucose tolerance	3 (5.5)	1 (1.0)	2 (3.7)	2 (6.7)	3 (3.7)	0.303
Primary hypothyroidism	5 (9.1)	8 (7.8)	6 (11.1)	0 (0)	7 (8.5)	0.449
Oral contraceptive use	2 (3.6)	8 (7.8)	0 (0)	0 (0)	6 (7.3)	0.242
Hyperprolactinemia	0 (0)	0 (0)	0 (0)	0 (0)	1 (1.2)	1.000
**Gastrointestinal diseases**						
Non-alcoholic fatty liver disease	19 (34.5)	17 (16.7)	10 (18.5)	5 (16.7)	24 (29.3)	0.203
Kinked gallbladder	7 (12.7)	12 (11.8)	3 (5.6)	3 (10.0)	3 (3.7)	0.053
Cholestasis	9 (16.4)	12 (11.8)	4 (7.4)	3 (10.0)	10 (12.2)	0.615
Cholelithiasis	4 (7.3)	3 (2.9)	1 (1.9)	1 (3.3)	5 (6.1)	0.572
Gilbert’s syndrome	2 (3.6)	0 (0)	0 (0)	0 (0)	1 (1.2)	0.170
Frequently recurring chronic pancreatitis	0 (0)	0 (0)	0 (0)	0 (0)	8 (9.8)	**<0.001** *p* _1–5_ = 0.023 *p*_2–5_ = 0.003 *p* _3–5_ = 0.025
**Other diseases and conditions**						
Malignant neoplasms	3 (5.5)	0 (0)	0 (0)	0 (0)	6 (7.3)	**0.007** *p* _1–2_ = 0.021 *p*_2–5_ = 0.008
Alcohol abuse	0 (0)	0 (0)	0 (0)	0 (0)	5 (6.1)	**0.010** *p* _2–5_ = 0.015

**Table 3:** Comparative analysis of clinical criteria among patients with FH and non-FH.

Indicator	FH, *n* = 241				Non-FH (Group 5, *n* = 82)	*p*
	Unlikely (Group 1, *n* = 55)	Possible (Group 2, *n* = 102)	Probable (Group 3, *n* = 54)	Certain (Group 4, *n* = 30)		
First-degree relatives with known premature CAD, *n* (%)						**0.001**
• yes	11 (20.0)	50 (49.0)	37 (68.5)	28 (93.3)	7 (8.5)	*p* _1–2_ = 0.002 *p*_1–3_ < 0.001 *p*_1–4_ < 0.001 *p*_1–5_ = 0.011 *p*_2–3_ = 0.022 *p*_2–4_ < 0.001 *p*_3–4_ = 0.011 *p*_3–5_ = 0.008 *p*_4–5_ < 0.001
• no	43 (78.2)	52 (51.0)	17 (31.5)	2 (6.7)	46 (56.1)	*p* _1–2_ = 0.001 *p*_1–3_ < 0.001 *p*_1–4_ < 0.001 *p*_2–3_ = 0.022 *p*_2–4_ < 0.001 *p*_2–5_ < 0.001 *p*_3–4_ = 0.011 *p*_3–5_ < 0.001 *p*_4–5_ < 0.001
• unknown	1 (1.8)	0 (0)	0 (0)	0 (0)	29 (35.4)	*p* _1–5_ < 0.001 *p*_2–5_ < 0.001 *p*_3–5_ < 0.001 *p*_4–5_ = 0.002
First-degree relatives or children with LDL-C levels above the 95th percentile for age and sex, *n* (%)						**<0.001**
• yes	18 (32.7)	40 (39.2)	31 (57.4)	17 (56.7)	11 (13.4)	*p* _1–3_ = 0.019 *p*_1–4_ = 0.046 *p*_1–5_ = 0.017 *p*_2–3_ = 0.046 *p*_2–5_ < 0.001 *p*_3–5_ < 0.001 *p*_4–5_ < 0.001
• no	35 (63.6)	35 (34.3)	5 (9.3)	2 (6.7)	39 (47.6)	*p* _1–2_ = 0.001 *p*_1–3_ < 0.001 *p*_1–4_ < 0.001 *p*_2–3_ = 0.001 *p*_2–4_ = 0.004 *p*_3–5_ < 0.001 *p*_4–5_ < 0.001
• unknown	2 (3.4)	27 (26.5)	18 (33.3)	11 (36.6)	32 (39.0)	*p* _1–2_ = 0.001 *p*_1–3_ < 0.001 *p*_1–4_ < 0.001 *p*_1–5_ < 0.001
The patient has premature development of coronary heart disease, *n* (%)	1 (1.8)	4 (3.9)	6 (11.1)	5 (16.7)	5 (6.1)	0.484
The patient has premature development of cerebral or peripheral atherosclerosis, *n* (%)	3 (12.5)	21 (53.8)	12 (57.1)	14 (63.6)	11 (28.2)	**<0.001** *p* _1–2_ = 0.005 *p*_1–3_ = 0.005 *p*_1–4_ = 0.003 *p*_2–5_ = 0.043 *p*_3–5_ = 0.046 *p*_4–5_ = 0.017
Tendon xanthomas, *n* (%)	0 (0)	0 (0)	6 (10.9)	22 (73.3)	1 (1.2)	**<0.001** *p* _1–3_ = 0.018 *p*_1–4_ < 0.001 *p*_2–3_ = 0.002 *p*_2–4_ < 0.001 *p*_3–4_ < 0.001 *p*_3–5_ = 0.011 *p*_4–5_ < 0.001
Eyelid xanthelasmas, *n* (%)	0 (0)	1 (0.9)	4 (7.3)	5 (16.7)	1 (1.2)	**<0.001** *p* _1–4_ = 0.005 *p*_2–4_ = 0.002 *p*_4–5_ = 0.004
Lipid arcus cornea in patients under 45 years of age, *n* (%)	0 (0)	0 (0)	1 (1.8)	5 (16.7)	0 (0)	**<0.001** *p* _1–4_ = 0.005 *p*_2–4_ < 0.001 *p*_3–4_ = 0.012 *p*_4–5_ < 0.001
Age of TC increase, years, Me [25; 75]	43.0 [38.0; 47.5]	33.0 [29.0; 43.0]	35.0 [22.5; 66.5]	29.0 [20.0; 33.0]	46.0 [34.5; 56.0]	0.160
Maximum TC level, mmol/L, Me [25; 75]	7.0 [6.9; 7.9]	8.1 [7.2; 9.2]	9.4 [8.2; 11.0]	11.0 [8.5; 13.5]	7.3 [6.5; 9.0]	**<0.001** *p* _1–2_ < 0.001 *p*_1–3_ < 0.001 *p*_1–4_ < 0.001 *p*_2–3_ = 0.005 *p*_2–4_ < 0.001 *p*_3–5_ < 0.001 *p*_4–5_ < 0.001
Tested during lipid-lowering therapy	17 (30.9)	38 (37.3)	25 (45.5)	17 (56.7)	29 (35.4)	0.225
DLCN, points	1.0 [1.0; 1.0]	3.0 [3.0; 4.0]	7.0 [6.0; 7.0]	11.5 [9.0; 15.0]	0 [0; 0]	**<0.001** *p* _1–2_ < 0.001 *p*_1–3_ < 0.001 *p*_1–4_ < 0.001 *p*_2–3_ < 0.001 *p*_2–4_ < 0.001 *p*_2–5_ < 0.001 *p*_3–5_ < 0.001 *p*_4–5_ < 0.001

Note: TC–total cholesterol, DLCN–Dutch Lipid Clinic Network.

## Data Availability

Raw data are available upon request from the corresponding author. These data are not publicly available due to privacy concerns.

## Supplementary Materials

The following supporting information can be downloaded at: https://www.mdpi.com/article/10.3390/ijms27031214/s1.

## Abbreviations

CHD	Coronary heart disease
CVD	Cardiovascular disease
DLCN	Dutch Lipid Clinic Network
FH	Familial hypercholesterolemia
LDL-C	Low-density lipoprotein cholesterol
Me	Median
Non-FH	Non-familial hypercholesterolemia
TC	Total cholesterol
T2DM	type 2 diabetes mellitus
TG	Triglyceride
