# Supplementary material for: Comorbidities and Molecular Genetics Status in Familial and Nonfamilial Hypercholesterolemia: A Single-Center Study
Source: Int J Mol Sci. 2026 Jan 25;27(3):1214. doi: 10.3390/ijms27031214 (PMC12898640; doi:10.3390/ijms27031214)
Supplement: Supplementary file 1 [file ijms-27-01214-s001.zip › ijms-4006452-supplementary.pdf]

Genetic testing

Table S1. The genetic variants identified in Western Siberia patients with a phenotype of Familial Hypercholesterolemia. MAF: minor allele frequency.

dbSNP ID	Position on chromosome (GRCh38)	Nucleotide substitution*	Amino acid substitution	MAF according to database GnomAD (3.2.1)	Clinical effect according to database ClinVar
***LDLR* gene**					
rs121908038	19:11113293	c.1202T>A	p.Leu401His	ND	Conflicting classifications of pathogenicity (P/LP/VUS)
rs137853964	19:11129602	c.2479G>A	p.Val827Ile	A=0.000456	Uncertain significance (VUS)
rs2147257524	19:11116909	c.1756T>C	p.Ser586Pro	C=0.000003	Likely pathogenic
rs28942078	19:11113376	c.1285G>A	p.Val429Met	A=0.0000107	Pathogenic
rs539080792	19:11221396	c.1009G>A	p.Glu337Lys	A=0.0000364	Conflicting Interpretations of Pathogenicity (VUS/B/LB)
rs570942190	19:11113337	c.1246C>T	p.Arg416Trp	T=0.000013	Pathogenic/Likely pathogenic
rs755757866	19:11110730	c.1019G>T	p.Cys340Phe	T=0.000007	Pathogenic/Likely pathogenic
rs761954844	19:11110697	c.986G>A	p.Cys329Tyr	A=0.0000043	Pathogenic/Likely pathogenic
rs879254566	19:11105440	c.534T>G	p.Asp178Glu	ND	Pathogenic/ Likely Pathogenic
rs879254721	19:11107496	c.922G>A	p.Glu308Lys	ND	Likely pathogenic
rs879254980	19:11116179	c.1672G>T	p.Glu558Ter	ND	Pathogenic
rs879255191	19:11128090	c.2389+5G>A	-	ND	Uncertain significance (VUS)
rs875989907	19:11106666	c.796G>A	p.Asp266Asn	A=0.000008	Pathogenic
rs879254769	19:11110765	c.1054T>C	p.Cys352Arg	ND	Likely Pathogenic
rs875989894	19:11213415	c.266G>C	p.Cys89Ser	ND	Not reported in ClinVar
ND	19:11222252	c.1123T>G	p.Tyr375Asp	ND	Likely Pathogenic
ND	19:11106679	c.809G>A	p.Cys270Tyr	ND	Likely Pathogenic
***APOB* gene**					
rs5742904	2:21006288	c.10580G>A	p.Arg3527Gln	T=0.0006903	Pathogenic
***LPL* gene**					
rs118204077	8:19955873	c.808C>T	p.Arg270Cys	C=0.000006572	Pathogenic

**LDLR* - NM_000527.5, *APOB*- NM_000384.3, *LPL*- NM_000237.3

Identification of Deletions and Duplications in the *LDLR* Gene by MLPA

Forty-two patients, without functionally significant point substitutions in lipid metabolism genes, were subjected to MLPA analysis to find possible structural changes (deletions or duplications) in the *LDLR* promoter and exons. This analysis revealed deletions in DNA samples from two unrelated patients. In the first case, deletion NM_000527.4:c.(67+1_68-1)_(1586+1_1587-1)del in a heterozygous state eliminated a region spanning exons 2 to 10. In the second case, the patient was a carrier of a deletion of exon 15 in the *LDLR* gene NM_000527.4:c.(2140+1_2141-1)_(2311+1_2312-1)del in a heterozygous state.
